# A 17-kDa Fragment of Lactoferrin Associates With the Termination of Inflammation and Peptides Within Promote Resolution

**DOI:** 10.3389/fimmu.2018.00644

**Published:** 2018-03-28

**Authors:** Aviv Lutaty, Soaad Soboh, Sagie Schif-Zuck, Orly Zeituni-Timor, Ran Rostoker, Malgorzata J. Podolska, Christine Schauer, Martin Herrmann, Luis E. Muñoz, Amiram Ariel

**Affiliations:** ^1^The Laboratory for Molecular Pathways in the Resolution of Inflammation, The Department of Biology, University of Haifa, Haifa, Israel; ^2^The Department of Human Biology, University of Haifa, Haifa, Israel; ^3^Friedrich-Alexander-University Erlangen-Nürnberg (FAU), Department of Internal Medicine 3—Rheumatology and Immunology, Universitätsklinikum Erlangen, Erlangen, Germany

**Keywords:** resolution of inflammation, lactoferrin, macrophages, efferocytosis, NETosis

## Abstract

During the resolution of inflammation, macrophages engulf apoptotic polymorphonuclear cells (PMN) and can accumulate large numbers of their corpses. Here, we report that resolution phase macrophages acquire the neutrophil-derived glycoprotein lactoferrin (Lf) and fragments thereof *in vivo* and *ex vivo*. During the onset and resolving phases of inflammation in murine peritonitis and bovine mastitis, Lf fragments of 15 and 17 kDa occurred in various body fluids, and the murine fragmentation, accumulation, and release were mediated initially by neutrophils and later by efferocytic macrophages. The 17-kDa fragment contained two bioactive tripeptides, FKD and FKE that promoted resolution phase macrophage conversion to a pro-resolving phenotype. This resulted in a reduction in peritoneal macrophage numbers and an increase in the CD11b^low^ subset of these cells. Moreover, FKE, but not FKD, peptides enhanced efferocytosis of apoptotic PMN, reduced TNFα and interleukin (IL)-6, and increased IL-10 secretion by lipopolysaccharide-stimulated macrophages *ex vivo*. In addition, FKE promoted neutrophil-mediated resolution at high concentrations (100 µM) by enhancing the formation of cytokine-scavenging aggregated NETs (tophi) at a low cellular density. Thus, PMN Lf is processed, acquired, and “recycled” by neutrophils and macrophages during inflammation resolution to generate fragments and peptides with paramount pro-resolving activities.

## Introduction

The clearance of apoptotic cells is essential for proper development, homeostasis, and the termination of immune responses (1, 2). The engulfment of apoptotic polymorphonuclear cells (PMN) by macrophages during the resolution of inflammation, in particular, is considered to be a hallmark and a major fate-determining event for these cells (3–6). The phagocytosis of apoptotic cells and phagolysosome maturation reportedly result in the degradation of the apoptotic cell content (7). However, neutrophil-derived defensins were preserved and transferred from engulfed apoptotic PMN to the phagolysosomes in the engulfing macrophage (8). Here, they limit the intracellular growth of *Mycobacterium tuberculosis*. It has been shown that CD11b^low^ macrophages emerge during the resolution of inflammation (9). These satiated phagocytes engulf high numbers of apoptotic PMN and consecutively migrate to spleen and inguinal lymph nodes (LN) (9).

Lactoferrin (Lf), an iron-binding glycoprotein from the transferrin family with a molecular weight of 78 kDa, is found in various body fluids like milk, colostrum, saliva, tears, mucus secretions, as well as in neutrophil secondary granules (10). Neutrophil degranulation is the main source of Lf in blood (11). Lf is endowed with a plethora of biological functions, including antimicrobial, antiviral, antiparasite, anti-inflammatory, and anticancer activities (12). Lf-binding proteins have been detected on the surfaces of various cells, including neutrophils, monocytes, and peritoneal macrophages (13, 14), as well as on the surfaces of various microorganisms (15). Lf is an important pro-resolving mediator when it is released from apoptotic neutrophils, *i.e*., it blocks the directed migration of neutrophils and eosinophils (16, 17). The region(s) of Lf responsible for its bactericidal properties is still elusive, although the primary sequences of human Lf and bovine Lf are known, and the molecular structure of this protein has been studied by X-ray crystallography. Numerous studies found that pepsin-digested Lf has a stronger antimicrobial activity than the native protein. A peptide fragment of Lf that exerts antimicrobial activity is referred to as lactoferricin (18). Komine et al. reported that cleavage by the neutrophil-borne serine proteases elastase and/or proteinase 3 of human or bovine Lf generated several bioactive oligopeptides (19, 20). One of these oligopeptides (PGQRDLLFKDSAL) promoted the secretion of pro-inflammatory cytokines and chemokines [interleukin (IL)-6, IL-8, tumor necrosis factor α (TNFα), and monocyte chemotactic protein-1 (MCP-1)] by bovine mammary epithelial cells, whereas its human homolog induced the production of IL-6, MCP-1, and IL-8. In addition, it activated nuclear factor kappa B (NF-κB) in human oral epithelial HSC-2 cells (20). An additional sequence (FKDCHLA) that also contains an FKD motif showed similar pro-inflammatory properties (20), whereas its bovine counterpart (FKECHLA) was inactive (19). Hence, Lf can be cleaved into bioactive peptides that display activities different from the established antibacterial properties.

Recent reports have indicated that the accumulation of neutrophils in high density *in vivo* or *in vitro* and their activation with monosodium urate (MSU) crystals leads to the generation of aggregated neutrophil extracellular traps (aggNETs) in a reactive oxygen species-dependent manner (21). These tophus-like structures promote the degradation of inflammatory cytokines through cleavage by associated serine proteases (21). However, especially in glandular tissues, aggNETs may carry the risk to occlude ducts and secondarily precipitate inflammatory reactions (22). Lf and adenosine triphosphate enhanced aggNETs formation in low-density neutrophil cultures (21). This suggests that Lf and serine proteases cooperate in the resolution of inflammation.

Here, we report that murine resolution phase macrophages contain truncated forms of Lf that are generated by aged/senescent PMN and are acquired following their engulfment. In turn, distinct shorter fragments of Lf are found in body fluids during the inflammatory and resolving phases of both murine peritonitis and bovine mastitis. A resolution-associated fragment of Lf contained two tripeptides that modulated *in vivo* pro-resolving properties of macrophages as well as the formation of aggNETs. These results suggest that macrophages acquire Lf from senescent PMN, process it to shorter bioactive peptides, and fragments thereof release them in a temporal manner. These peptides in turn promote pro-resolving actions of neutrophils and macrophages, as needed.

## Materials and Methods

### Reagents

The following reagents were purchased as detailed: acrylamide/bis-acrylamide, fibronectin, lipopolysaccharide (LPS) (from *Escherichia coli*, clone 055:B5), staurosporine, TEMED, Tween-20, the neutrophil elastase inhibitor silevestat, and zymosan A from Sigma-Aldrich; clodronate from Liposoma BV; anti-goat horseradish peroxidase-conjugated immunoglobulin G (IgG) and anti-rabbit horseradish peroxidase-conjugated IgG from Jackson Immuno Research Laboratories; WesternBright™ ECL from Advansta, fetal calf serum (FCS), L-glutamine, penicillin–streptomycin, RPMI 1640 and trypan blue from Biological Industries, Kibbutz Beit Haemek; goat anti-mouse CD11b (M-19) polyclonal IgG and rabbit anti-human Lf (H-65) polyclonal IgG from SantaCruz Biotechnology; FITC-conjugated anti-mouse Gr-1, PE-conjugated anti-mouse F4/80, PerCP-conjugated anti-mouse CD11b, APC-conjugated anti-mouse CXCR4 (clone BL6-146508) and enzyme-linked immunosorbent assay (ELISA) kits for TNFα, IL-6, IL-12, and IL-10 from Biolegend; cycloheximide (CHX) from Cayman Chemical; PE selection kit was purchased from Stem Cell Technologies; and Protease Inhibitors Cocktail was purchased from Roche. FKD and FKE peptides were organically synthesized by GL Biochem (Shanghai) Ltd.

### Murine Peritonitis

Male C57BL/6 mice (6–8 weeks from Harlan Biotech, protocol approved by the Committee of Ethics, The Technion, authorization no. IL-065-04-2010) were injected intraperitoneally (i.p.) with sterile zymosan A (1 mg/ml in PBS). At 24 or 66 h post-peritonitis initiation (PPI), mice were euthanized using isoflurane, and peritoneal exudates were collected by lavaging with 5 ml of sterile PBS. In some experiments, mice challenged with zymosan A for 42 h or unchallenged were injected i.p. with pre-made liposomal clodronate (1 or 0.1 mg/mouse, respectively) or empty liposomes. Then, unchallenged mice were injected with zymosan A. After additional 24 h, peritoneal fluids and spleens were collected from all mice and analyzed for leukocyte subsets by flow cytometry and by Western blotting for Lf as indicated below. Clodronate treatment resulted in an 84% reduction in peritoneal macrophages and no significant changes in the percentage of splenic macrophages. In other experiments, vehicle, FKD, or FKE peptides (50 µM) were injected i.p. at 48 h PPI, and peritoneal exudates were recovered at 66 h. Macrophages from the exudates were evaluated for CD11b expression, their apoptotic PMN content, and reprogrammed cytokine secretion as detailed below.

### Apoptosis Induction

Jurkat T cells (1 × 10^6 ^cell/ml) were cultured in culture media (RPMI 1640 supplemented with 10% FCS, 2 mM L-glutamine, 100 µg/ml streptomycin, and 100 U/ml penicillin) under a humidified 5% CO_2_ atmosphere at 37°C. Apoptosis was induced by staurosporine (1 µM) for 4 h. PMN cells were isolated from peritoneal exudates 24 h PPI and incubated (1 × 10^6^ cell/ml) in culture media under a humidified 5% CO_2_ atmosphere at 37°C for 16–24 h or for 4 h with roscovitine (10 µM) to promote apoptosis. After culturing, each cell type was collected, and the percentage of apoptotic cells was detected using MEBCYTO apoptosis kit (MBL). In some experiments, PMNs were treated with protease inhibitor (diluted 1:25), CHX (30 mM), or silevestat (40 nM), concomitantly with roscovitine and then analyzed for Lf content by Western blotting. In some experiments, the neutrophils were stained for CXCR4 and Annexin V and analyzed by flow cytometry.

### Apoptotic Cell Engulfment *Ex Vivo*

Neutrophils or macrophages were isolated from peritoneal exudates 24 or 66 h after zymosan A injection, respectively, using EasySep^®^ Selection Kit following the manufacturer’s instructions (Stem Cells Technologies Inc.) with primary antibodies directed against Ly-6G and F4/80, respectively. Then, macrophages were incubated with either apoptotic Jurkat T cells or senescent PMNs (1:5 ratio) for 24 h. Next, unbound cells were washed with PBS and the adherent cells were lysed with radioimmunoprecipitation assay (RIPA) buffer containing protease inhibitor cocktail for 20 min on ice, followed by centrifugation (15,000 RPM, 4°C) for 15 min and collection of supernatants.

### Interstitial Fluids (ISFs) From Lymphoid Organs

Inguinal LN and spleens were harvested from mice 24 or 66 h PPI. The organs were mechanically dissociated and strained through a 100-µm nylon mesh (Beckton-Dickinson) to produce a single cell suspension, followed by separation of cells from ISF by centrifugation (1,200 RPM for 5 min). Then, red blood cells were lysed using RBC Lysis Buffer (biological industries), and both fluids and cells were saved at −20°C for further analysis. ISFs were added with a sample buffer and analyzed by Western blotting for Lf.

### Mastitis Milk Sample Preparation

Milk samples were obtained from three different dairy cow farms. Samples were taken from cows undergoing resolving or non-resolving *E. coli*-induced mastitis or from healthy cows based on farmer’s criteria. The farmers’ criteria for resolving mastitis were high somatic cell counts and a swollen and red udder that resolved to a healthy state within 7–10 days. The milk initially became watered-down but later restored normal complexion. Non-resolving mastitis had the same diagnosis parameters initially, but instead of resolving, the milk became bloody and the udder became painful upon touching and eventually clogged. In order to separate the cellular and fat fraction from the aqueous fraction, samples were centrifuged at 1,200 RPM for 5 min (cellular fraction), and the supernatants were incubated overnight at 4°C to solidify the fat fraction. Then, the fat fraction was mechanically peeled from samples and sample buffer was added to the samples. Equal milk volumes were analyzed by Western blotting for Lf.

### Purification of Lf-Derived Fragments

Mastitis milk samples from day 5 of resolving mastitis were defatted by centrifugation at 2,000 × *g* for 30 min at 4°C. The pH of the skim milk was adjusted to 4.6 with 5 N HCl and the milk was centrifuged at 10,000 × *g* for 1 h to remove the casein precipitate. The whey was then passed through a 0.45-mm filter (Millipore) to completely remove the casein precipitate and its pH was readjusted to 6.0 with 1 N NaOH. The immunoglobulin in the whey was removed by ammonium sulfate precipitation (48%). After passing through a 0.45-mm filter, the solution in the whey was replaced with 0.005 M sodium phosphate buffer (pH 6.0) using dialysis bag (33 mm cellulose membrane, 12–14 kDa cutoff, Sigma-Aldrich). Dialysis was performed in two repetitions, for 5 h and consequently overnight with stirring in a high volume of 0.005 M sodium phosphate buffer (pH 6.0) at 4°C. Then, the samples were loaded into a heparin affinity column (Affi-gel Heparin gel, Bio-Rad), washed and eluted by a stepwise elution with increasing salt concentrations (0.005 M sodium phosphate buffer; pH 6.0, containing 0.1, 0.3, or 0.5 M NaCl). The proteins were then collected at the 0.5 M NaCl fraction, and its content was assessed by sodium dodecyl sulfate-polyacrylamide gel electrophoresis (SDS-PAGE) followed by Coomassie Blue staining and Western blotting for Lf (SantaCruz).

### Western Blotting

Isolated cells were washed with PBS and lysed in RIPA buffer containing Protease Inhibitors Cocktail (1:25 dilution, Roche). Cell lysates, ISF, milk samples or isolated Lf derivatives were added with a sample buffer, run by SDS-PAGE (7.5 or 10%), and then transferred to a polyvinylidene difluoride membrane. The membranes were blocked with 5% BSA and probed with primary rabbit anti-mouse Lf polyclonal IgG (SantaCruz). Next, the membranes were blotted with appropriate secondary antibodies (1:10,000 dilution, 1 h at room temperature, Jackson ImmunoResearch) conjugated with horseradish peroxidase. Membranes were developed with WesternBright™ ECL kit (Advansta) and analyzed using Luminescent Image Analyzer LAS-4000 (Fujifilm Corporation) and “Image Reader LAS-4000” software (Fujifilm Corporation). Densitometric analysis was performed using TotalLab TL100 (nonlinear dynamics) image analysis software.

### Lf Fragment Sequencing

The Coomassie-stained bands corresponding to the 15- and 17-kDa fragments of bovine Lf from milk samples were proteomically analyzed at the Smoler Proteomics Center, The Technion, Israel, according to the following protocol: the proteins in each sample were denatured in 8 M urea, reduced with 3 mM DTT (60°C for 30 min), and modified with 10 mM iodoacetamide in 100 mM ammonium bicarbonate (room temperature for 30 min). The urea was diluted to 2 M, and the sample was trypsinized in 10 mM ammonium bicarbonate containing trypsin (modified trypsin; Promega) at a 1:50 enzyme-to-substrate ratio, overnight at 37°C. A second step of trypsinization was performed by adding another portion of trypsin and incubation at 37°C for 4 h. Mass spectrometry was done according to the following protocol: the resulting peptides were desalted using C18 tips (homemade), dried, and re-suspended in 50 mM Hepes (pH 6.4). Labeling by dimethylation was done in the presence of 100 mM NaCBH_3_ (Sterogene 1 M), by adding light formaldehyde (35% Frutarom, 12.3 M) to one of the samples and heavy formaldehyde (20% w/w, Cambridge Isotope Laboratories, 6.5 M) to the other sample to a final concentration of 200 mM. After 1-h incubation at room temperature, the pH was raised to 8 and the reaction was incubated for 1 h. Neutralization was done with 25 mM ABC for 30 min, and equal amounts of the light and heavy peptides were mixed, cleaned on C18, and re-suspended in 0.1% formic acid. The peptides were resolved by reverse-phase chromatography on 0.075 × 200-mm fused silica capillaries (J&W) packed with Reprosil reversed phase material (Dr. Maisch GmbH, Germany). The peptides were eluted with linear 90-min, gradients of 5–45% and 15 min at 95% acetonitrile with 0.1% formic acid in water at flow rates of 0.25 µl/min. Mass spectrometry was performed by an ion-trap mass spectrometer (Orbitrap, Thermo) in a positive mode using repetitively full MS scan followed by collision-induced dissociation (CID) of the seven most dominant ions selected from the first MS scan.

The mass spectrometry data were analyzed using the Sequest 3.31 software (J. Eng and J. Yates, University of Washington and Finnigan, San Jose) searching against the bovine part of the NCBI-NR database. Peptide quantities were calculated as area under the pick using the PepQuant algorithm from Bioworks.

### Sequence Alignment

Sequences from the identified peptides were aligned relative to full-length Lf using STRAP, Interactive Structure based Sequence Alignment Program,^1^ along with PubMed protein database.^2^ For an illustration of the complete isolation and sequencing procedure, please see Figure S2 in Supplementary Material.

### Macrophage CD11b Expression

Peritoneal exudates were recovered from mice 66 h PPI following treatment for 18 h with vehicle, FKD, or FKE peptides. The cells were enumerated and immune-stained for Gr-1, F4/80, and CD11b and analyzed by flow cytometry (FACSCanto II) for leukocyte subtypes. F4/80^+^ macrophages were analyzed for the expression of CD11b as previously (9).

### *In Vivo* Efferocytosis

Peritoneal macrophages from Section “Macrophage CD11b Expression” were isolated using F4/80-directed magnetic beads, and 150 × 10^3^ cells were plated in an 8-well glass chamber slide (Nunc) for 2 h in 150-µl culture medium. Then, medium was aspirated, and unbound cells were washed gently with PBS. Adherent cells were subsequently fixed (200 µl of 4% paraformaldehyde, 5% sucrose, 15 min). Next, the cells were washed in PBS and stained (overnight, 4°C) with 200 µl of CF488A-conjugated phalloidin (5 U/ml from Biotium), for F-actin. Next, cells were washed three times with PBS and stained for nuclear DNA in 200 µl of Hoechst (20 µg/ml, 5 min, from Invitrogen), and rinsed with PBS. Then, the chambers were removed, mounted with Fluoromount G, and visualized under a Nikon A1 confocal microscope. The number of engulfed apoptotic cells per macrophage was scored, as well as the percentage of non-engulfing macrophages. The results were obtained from eight mice and 503–592 macrophages per treatment.

### Macrophage Cytokine Secretion

Peritoneal macrophages from Section “Macrophage CD11b Expression” were isolated and then treated with LPS (1 µg/ml, 24 h) or vehicle. Then, culture media were collected and its cytokine content was determined by standard ELISA for TNFα, IL-6, IL-10, or IL-12.

### Regulation of aggNETs Formation

All analyses of material derived from human subjects were performed in full agreement with institutional guidelines and with the approval of the Ethical Committee of the University Hospital Erlangen (permit number 193 13B). Peripheral blood PMNs (>95% neutrophils) were separated from mononuclear cells by density gradient centrifugation on Lymphoflot (Bio-Rad, Hercules, CA, USA) and cleared from contaminating erythrocytes by short hypotonic lysis. PMNs were incubated at a low cell density (5 × 10^6^ cells/ml) with 50 pg/cell MSU crystals and/or 10–100 µM Lf (Sigma-Aldrich) and/or 10–100 µM FKD or FKE peptides and 1 µg/ml propidium iodide solution in RPMI (Thermo Fisher Scientific) without serum supplementation for 3 h at 37°C and 5% CO_2_. NET aggregation was stopped by adding 1% paraformaldehyde to cell cultures for 20 min. NET aggregates were filtrated through a 40-µM mesh, and macrophotographs of captured material with UVB transillumination were made with a Nikon D700 reflex camera.

### Data Analysis

Experimental data were analyzed by Student’s *t*-test or ANOVA followed by Tukey’s *post hoc* analysis. *p*-values of ≤0.05 were designated as statistically significant. Results are presented as average ± SEM.

## Results

### Resolution Phase Macrophages Acquire Lf From Senescent PMN

**Figure 1:**
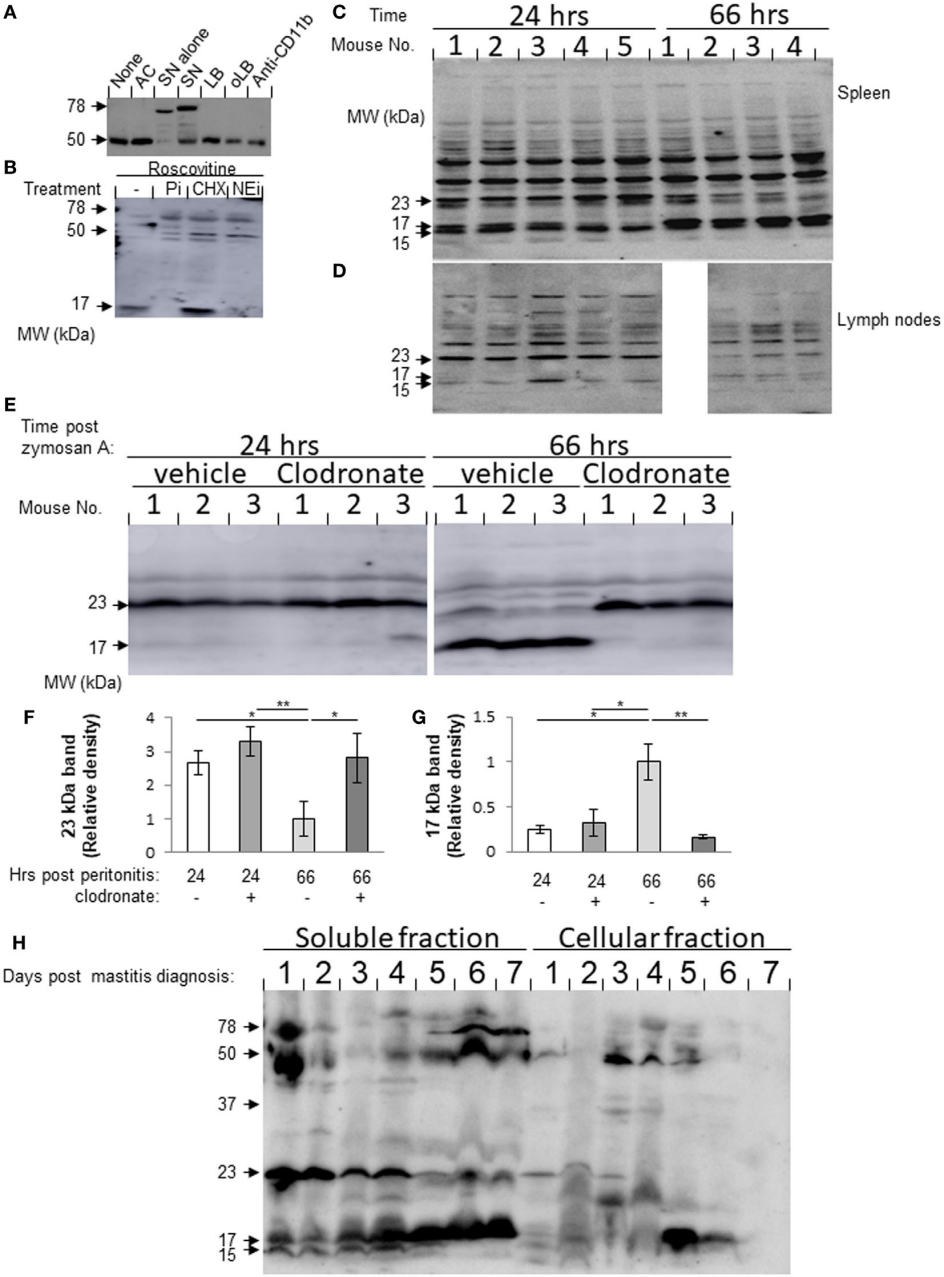
**(A,B)** Lactoferrin (Lf) fragments accumulate in macrophages following incubation with senescent neutrophils. **(A)** Peritoneal macrophages were recovered 66 h post zymosan A-induced (1 mg/mouse) peritonitis initiation and incubated with apoptotic Jurkat cells (AC), senescent peritoneal neutrophils (SN), latex beads (LBs), IgG-opsonized LB (oLB), or anti-CD11b monoclonal antibodies as indicated. After 24 h, unbound cells were washed and macrophages were recovered. Then, the cells were lysed, and equal amounts of protein extract were blotted for Lf. Protein extracts from apoptotic neutrophils were also analyzed as indicated. Results are representative from three independent experiments (cells were pooled from three to five mice). **(B)** Peritoneal neutrophils were recovered 24 h PPI and treated with roscovitine (10 µM) alone or with protease inhibitor cocktail (Pi), cycloheximide (CHX), or the neutrophil elastase inhibitor, silevestat (NEi) for 20 h. Then, neutrophils were lysed, and their protein content was immunoblotted for Lf [results are representative from three experiments (cells were pooled from three to five mice)]. **(C,D)** Lf fragments are found in interstitial fluids (ISFs) of spleen and inguinal lymph nodes (LN) during peritonitis. Spleen **(C)** and inguinal LN **(D)** were harvested 24 and 66 h after initiation of peritonitis (*n* = 4–5) and mashed in equal volumes of PBS employing a nylon grid. Equal amounts of ISF protein were run by 10% sodium dodecyl sulfate-polyacrylamide gel electrophoresis (SDS–PAGE), followed by Western blotting for Lf. Results are representative blots from three experiments. **(E–G)** Peritoneal resolution phase macrophages are essential for the differential production of splenic Lf fragments. Mice were injected intraperitoneally (i.p.) with clodronate-containing or empty liposomes 42 h PPI or before the initiation of peritonitis. After additional 24 h, the spleens were recovered and mashed in equal volumes of PBS employing a nylon grid. Equal amounts of ISF protein were run by 12% SDS-PAGE, followed by Western blotting for Lf. Results are a representative blot **(E)** and averages of densitometric analysis of the 23- **(F)** and 17 **(G)**-kDa bands from three experiments (*n* = 3–6 mice per group). **/*indicate statistically significant differences of *P* ≤ 0.01/*P* ≤ 0.05, respectively, by ANOVA with Tukey *post hoc* analysis. **(H)** Lf fragments are found in dairy cow milk during the onset and resolution of mastitis. Milk samples from dairy cows with mastitis (days 1–7) were defatted and separated into soluble and cellular fractions by centrifugation. The cellular fraction was lysed and the cytoplasmic proteins were recovered. Equal amounts of protein from both fractions were analyzed by Western blotting for Lf. Results show representative blots from three sample sets taken daily from three different cows.

During the resolution of inflammation, macrophages engulf apoptotic PMN and consecutively migrate to lymphoid organs (9, 23). Macrophages acquire granular defensins from neutrophils that had undergone apoptosis and “recycle” it for antimicrobial activity (8). Lf is a major constituent of neutrophils secondary granules. We hypothesized that Lf is acquired by macrophages once they engulf senescent PMN. To test this, we incubated neutrophils with resolution phase macrophages from the peritoneum *ex vivo* and quantified their levels of Lf. Apoptotic Jurkat T cells, latex beads (LBs), IgG-opsonized LB (oLB), and anti-CD11b antibodies were used as phagocytosis and CD11b crosslinking controls, respectively. The results in Figure 1A show that macrophages contained only a single 50-kDa band immunoreactive with anti-Lf antibodies. Incubation with aging/senescent neutrophils resulted in the appearance of an additional 78-kDa protein similar to the major band corresponding to neutrophil-borne Lf. Interestingly, the expression of the 50-kDa band in macrophages was not significantly modulated by incubation with apoptotic Jurkat cells or other phagocytic targets, such as LB and oLB. Crosslinking of CD11b did not modulate Lf levels in resolution phase macrophages either. To determine whether Lf fragmentation already takes place in PMN or whether Lf is expressed *de novo* in PMN, the recovered cells were treated with roscovitine in the presence of a general protease inhibitor cocktail, an elastase inhibitor (silevestat), or CHX. The results in Figure 1B indicate that roscovitine treatment enhanced Lf fragmentation to shorter fragments than 50 kDa already in unengulfed PMN. A fragment of 17 kDa was especially evident in apoptotic PMN. Moreover, both protease inhibitors significantly abrogated the generation of the 17-kDa Lf fragment, whereas CHX increased this Lf fragment levels. It was previously shown that senescent neutrophils express CXCR4 and migrate to the BM once they age through CXCL12 gradients (24). Of interest, the aging of peritoneal neutrophils *ex vivo* resulted in membrane expression of both annexin V and CXCR4 on most cells after 24 h (Figure S1 in Supplementary Material), and this was enhanced by roscovitine at 4 h, but not at 24 h. This indicates that aging PMNs have features of both apoptotic and senescent neutrophils. Altogether, these results indicate that Lf was processed, at least partially, in neutrophils during the apoptotic process, and consequently Lf and its fragments were acquired by resolution phase macrophages following the phagocytosis of apoptotic cells. Thus, macrophages can acquire Lf and its fragments from senescing neutrophils following their engulfment and maintain it as distinct fragments.

### Resolution-Associated Fragments of Lf Are Present in Murine Draining LN and Spleen as Well as Bovine Udder

Next, we analyzed whether Lf fragments can be detected in lymphoid tissues during inflammation and its resolution, since macrophages that engulf apoptotic PMN tend to adopt the CD11b^low^ phenotype and migrate to lymphoid organs (9). To this end, we collected ISFs from meshed inguinal LN and spleens 24 and 66 h post initiation of murine peritonitis. Our results in Figures 1C,D show three specific bands corresponding to Lf fragments that were detected in both spleen and LN with molecular weights of 23, 17 and 15 kDa. Notably, the 23-kDa fragment was present at a higher amount during the inflammatory phase of the response (24 h) in comparison to the resolution phase (66 h) in both organs. The 15-kDa fragment showed a similar trend in the LN but did not reduce during the resolution phase in the spleen. Importantly, the 17-kDa fragment of Lf showed the opposite trend in both spleen and LNs. Essentially, its levels were significantly increased during the resolution phase compared to the inflammatory phase of peritonitis. To determine whether processing and/or release by peritoneal macrophages is key to determining the different fragments of Lf present at the spleen, we depleted peritoneal macrophages using clodronate 24 h prior to spleen recovery. Our results (Figures 1E–G) indicate that the depletion of resident macrophages did not affect the levels of either the 23- or 17-kDa fragments of Lf in the ISF at 24 h PPI. However, the depletion of resolution phase macrophages significantly reduced the levels of the 17-kDa fragment and increased the levels of the 23-kDa fragment at 66 h PPI. Thus, our results suggest that the inflammatory 23-kDa fragment of Lf is produced primarily by other cells, while the resolution phase-associated 17-kDa fragment is acquired and released by peritoneal macrophages that engulf PMN and migrate to the spleen.

Lactoferrin is a major component of milk and plays a key role in innate immune defense. We aimed to determine whether Lf fragments are also present in bovine milk during mastitis and its resolution, since neutrophils and macrophages increased in bovine milk during inflammation (25, 26). Our previous results determined that Lf fragments are accumulating in macrophages. Therefore, we sought to determine whether Lf fragments are also present in bovine milk during mastitis and its resolution. To this end, protein samples from the soluble and cellular fractions of bovine milk were collected daily during resolving mastitis (Figure S2 in Supplementary Material for Methodology; Figure 1H). As controls, milk samples were also collected from non-resolving mastitis as well as from healthy cows (Figure S3 in Supplementary Material). Our results indicate a similar fragmentation pattern for murine and bovine Lf. Moreover, during spontaneous resolution of mastitis, the amounts of the 17-kDa fragment of soluble Lf increase from day 1 until day 7. Concomitantly, the amounts of the 23- and 15-kDa fragments of Lf were decreased during the transition from the inflammatory phase to the resolving phase. The full-length 78-kDa Lf and its 50-kDa fragment are evident only at the beginning of the inflammatory episode (day 1) and during the late resolution phase of mastitis (days 6 and 7), while during the course of early inflammation, its levels are very low. Importantly, the 50- and 17-kDa fragments of Lf were also found in the cellular fraction of the milk from resolving mastitis, albeit in kinetics that preceded the soluble fraction by 24 h. This suggested that the soluble fragments originated in leukocytes that infiltrated into the infected udder. In non-resolving mastitis, the prevalence of Lf fragments was different from that of resolving mastitis. Only the 50-kDa fragment is evident in non-resolving mastitis, and its levels in the milk increased with time. Altogether, our findings indicate that Lf undergoes a distinct proteolytic processing during inflammation and its resolution in various organs and mammals, and that a leukocyte-derived 17-kDa fragment accumulates in various immune-active organs during the resolution of inflammation.

### Isolation of Lf-Derived Fragments and Determination of Their Amino Acid Sequences

**Figure 2:**
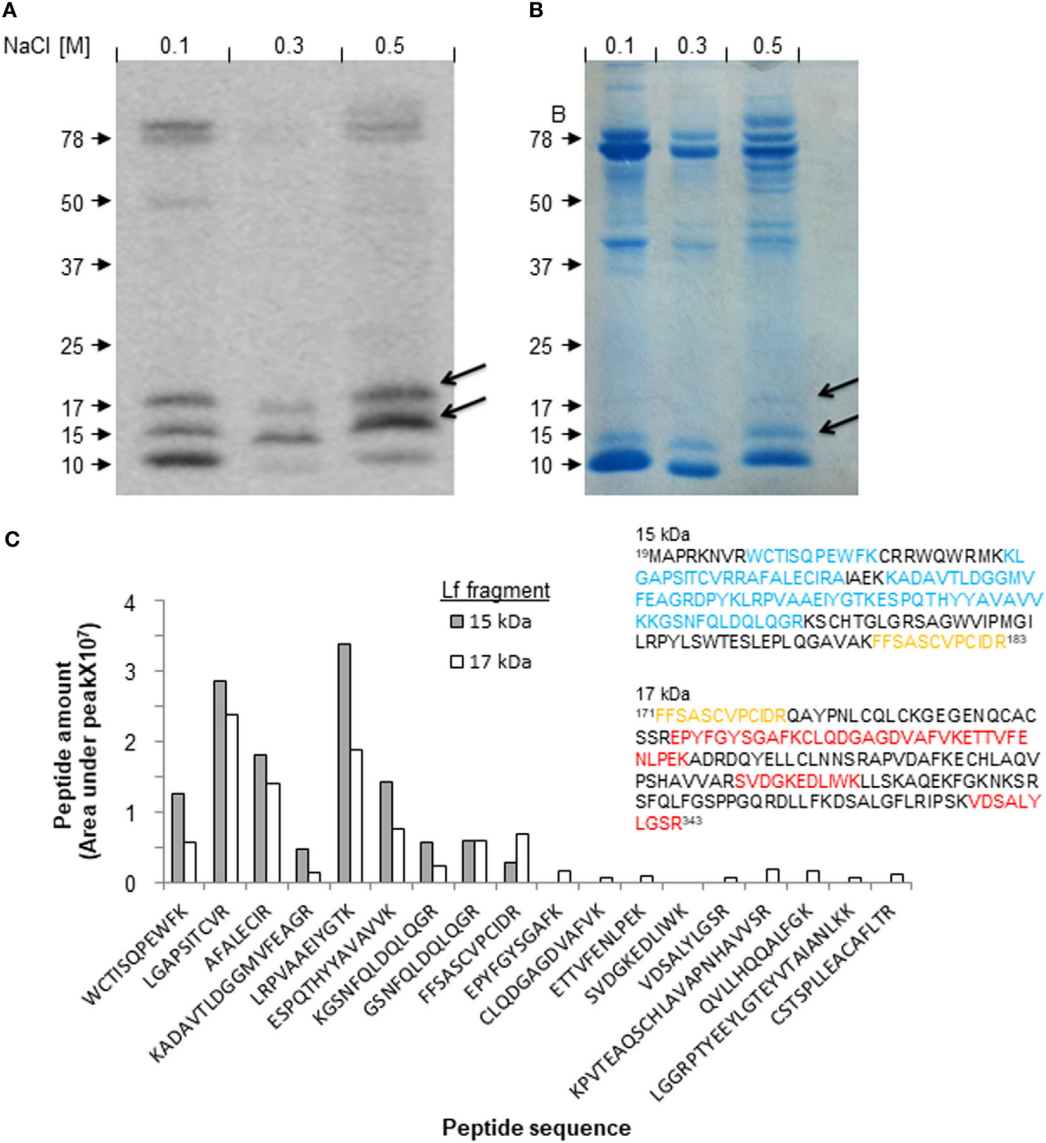
Isolation and identification of lactoferrin (Lf)-derived fragments from udders with resolving inflammation. Milk samples from cows with mastitis (day 5, early phase of resolution) were defatted and run on heparin gel columns. The bound proteins and fragments were eluted stepwise with 0.005 M sodium phosphate buffer (pH 6.0) containing 0.1, 0.3, or 0.5 M NaCl. The recovered protein samples were run on 10% sodium dodecyl sulfate-polyacrylamide gel electrophoresis (SDS-PAGE) followed with Western blotting for Lf **(A)**, and Coomassie Brilliant Blue staining for total protein content **(B)**. The bands corresponding to the 15- and 17-kDa fragments of Lf were excised from the gel and analyzed by LC-MS-MS. Quantification of the frequency of each fragment was provided by analysis of area under the peak. The indicated fragments were aligned using STRAP software against the full-length Lf sequence taken from the PubMed database. Then, each peptide was associated with the corresponding fragment of 15 or 17 kDa (inset, highlighted in blue and red, respectively). One common peptide present in both fragments is highlighted in orange **(C)**.

Next, we isolated the 17-kDa fragment of Lf from milk collected during the resolution of mastitis and determined its amino acid sequence, to identify novel resolution-associated molecules. To this end, milk samples from day 5 of mastitis were prepared and loaded on heparin liquid-gel columns. After washing of unbound material, the eluted products were run on 10% SDS-PAGE, blotted by anti-Lf antibodies, and visualized by Coomassie Brilliant Blue staining (Figures 2A,B). The bands at 17 and 15 kDa were identified as Lf fragments by Western blotting and therefore were excised from the gel and used for amino acid sequencing using mass spectrometry. No other small Lf-derived fragments were detected in our isolated proteins, suggesting that the other fragments found in bovine milk do not bind heparin. The proteomic analysis of the 15- and 17-kDa fragments of Lf resulted in a series of peptides with relative quantities (Figure 2C) that were aligned to the amino acid sequence of full-length bovine Lf as well as to Lf sequences from eight other species taken from the PubMed database. These species include *Homo sapiens, Mus musculus*, and two subgroups of the bovine family: *Bos indicus* and *Bos taurus*. Alignment, amino acid coloring, frame shift, and amino acid numbering were done using the STRAP software (Figure S4 in Supplementary Material). Our analysis was based on the notion that our anti-Lf antibody was generated against a peptide comprising amino acids 146–210, and therefore this portion of Lf should be included in both the 15- and 17-kDa fragments. Our results revealed that the 15-kDa fragment was composed of eight peptides that encompassed amino acids 19–183 (including the peptides ^27^WCTISQPEWFK^38^,^47^ KLGAPSITCVRRAFALECIRA^68^, ^72^KADAVTLD^80^, ^80^GGMVFEAGRD^90^,^90^ PYKLRPVAAE^100^,^100^ IYGTKESPQT^110^, ^110^HYYAVAVVKK^120^, ^120^GSNFQLDQLQGR^132^, and ^171^FFSASCVPCIDR^183^). Most of these peptides also appeared with similar frequencies in the 17-kDa fragment analysis (probably due to dispersion of the 15-kDa band and inaccuracies in the excision process). However, a second molecular species was also evident. This fragment ranged from amino acids 171–343 (including the peptides ^171^FFSASCVPCIDR^183^, ^206^EPYFGYSGAF^216^, ^217^CLQDGAGDVAFVK^230^, ^231^ETTVFENLPEK^241^, ^278^SVDGKEDLIWK^289^, and ^333^VDSALYLGSR^343^; Figure 2C, insert). These designated amino acid sequences matched in predicted molecular weights to 15 and 17 kDa, respectively. Notably, another Lf peptide that was not detected by Western blotting was identified using mass spectrometry within the 17-kDa band. This fragment from the C-lobe of Lf (containing peptides ^598^PVTEAQSCHLAVAPNHAVVSR^619^, ^627^QVLLHQQALFGK^639^, ^669^LGGRPTYEEYL-GTEYVTAIANLKK^693^, and ^693^CSTSPLLEACAFLTR^708^) was not further pursued as it is not known whether it is associated exclusively with the resolution phase of inflammation. Thus, we conclude that the 15- and 17-kDa fragments differ from one another, for the most part, with a short overlapping region in the domain that is recognized by the anti-Lf antibodies (Figure 2C, highlighted in orange).

### The Lf-Derived Peptides FKE and FKD Promote *In Vivo* Macrophage Conversion to the Pro-Resolving CD11b^low^ Phenotype

**Figure 3:**
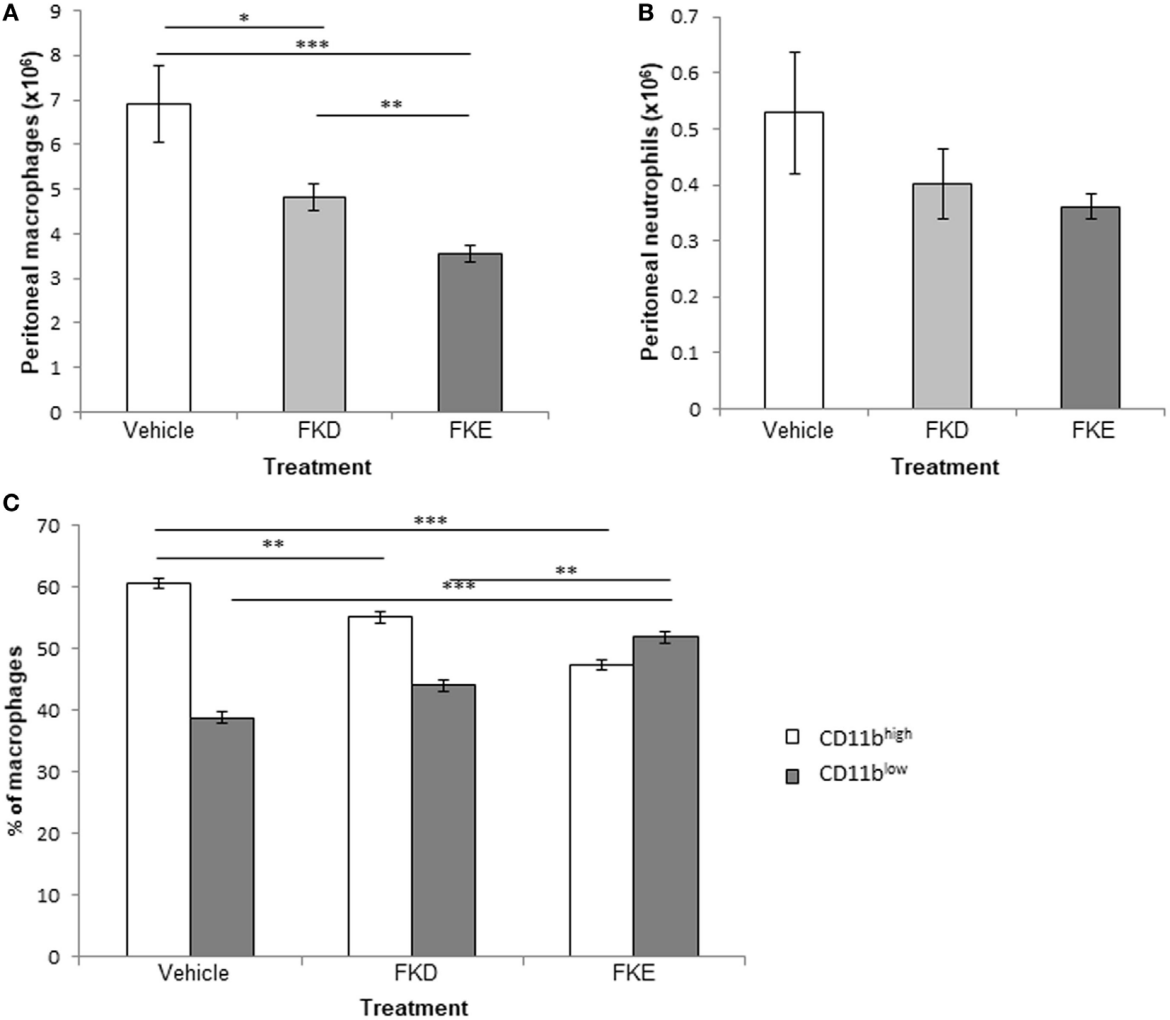
Lactoferrin (Lf)-derived peptides promote macrophage conversion to the CD11b^low^ phenotype. Mice undergoing peritonitis for 48 h were injected intraperitoneally (i.p.) with PBS, FKE, or FKD peptides (50 µM each). After additional 18 h, peritoneal exudates were recovered and the cells were enumerated. Then, exudate cells were immunostained for Ly-6G, F4/80, and CD11b and analyzed by flow cytometry. The numbers of macrophages **(A)** and neutrophils **(B)**, and the percentages of CD11b^high^ and CD11b^low^ macrophages **(C)** are presented. Results are averages ± SEM from three independent experiments (*n* = 4 per group). ***/**/*indicate statistically significant differences of *P* ≤ 0.005/*P* ≤ 0.01/*P* ≤ 0.05, respectively, by ANOVA with Tukey *post hoc* analysis.

Previously, Komine et al. (19) identified four peptides in serine protease-digested bovine Lf: FKECHLA, VPSHAVVAR, FQLFGSP, and PGQRDLLFKDSAL (designated pep1–4, respectively). All peptides are located within the 17-kDa Lf fragment (Figure S4 in Supplementary Material, underlined) and showed that pep4 had an enhanced pro-inflammatory effect in terms of cytokine and chemokine secretion in a bovine mammary gland epithelial cell line (19), while pep1–3 were devoid of activity. Other results from the same group (20) showed that the human homolog of pep1 contains an FKDCHLA sequence, induced pro-inflammatory cytokines and chemokines (IL-8, IL-6, and MCP-1), and activated NF-κB in the HSC-2 cell line. The human homolog of pep 4 (SGQKDLLFKDSAI) was active as well. Thus, we suggest that the difference in immune-modulatory properties between bovine pep4 and pep1 could be due to the replacement of a single aspartate by glutamate (D → E). As a result, the active consensus sequence changes from phenylalanine–lysine–aspartate (FKD) to phenylalanine–lysine–glutamate (FKE; Figure S4 in Supplementary Material, inside the red frame) and results in loss of activity under their experimental setting. Since our results indicate that both peptides are present in the 17-kDa Lf-derived fragment that accumulates during the resolution of inflammation in various species and models, we determined whether these peptides exert anti-inflammatory and pro-resolving actions and whether the FKD and FKE peptides differ in this respect. To this end, FKD and FKE peptides (50 µM) were injected i.p. to mice undergoing peritonitis for 48 h. After additional 18 h, peritoneal exudates from these mice were recovered, and the properties of the leukocytes collected were determined. Our results (Figures 3A,B) indicate that FKD, and to a higher extent FKE, reduced peritoneal macrophage and PMN numbers (0.7- and 0.51-fold of PBS for macrophages, 0.77- and 0.69-fold of control for PMN, respectively), although the latter reduction was not statistically significant. Importantly, the conversion of macrophages from the reparative CD11b^high^ phenotype to the pro-resolving CD11b^low^ phenotype (9, 27) in the peritoneum was upregulated by FKD and to a higher extent by FKE peptides (Figure 3C; 1.13- and 1.33-fold of PBS, respectively).

### FKE Peptides Promote Macrophage Efferocytosis

**Figure 4:**
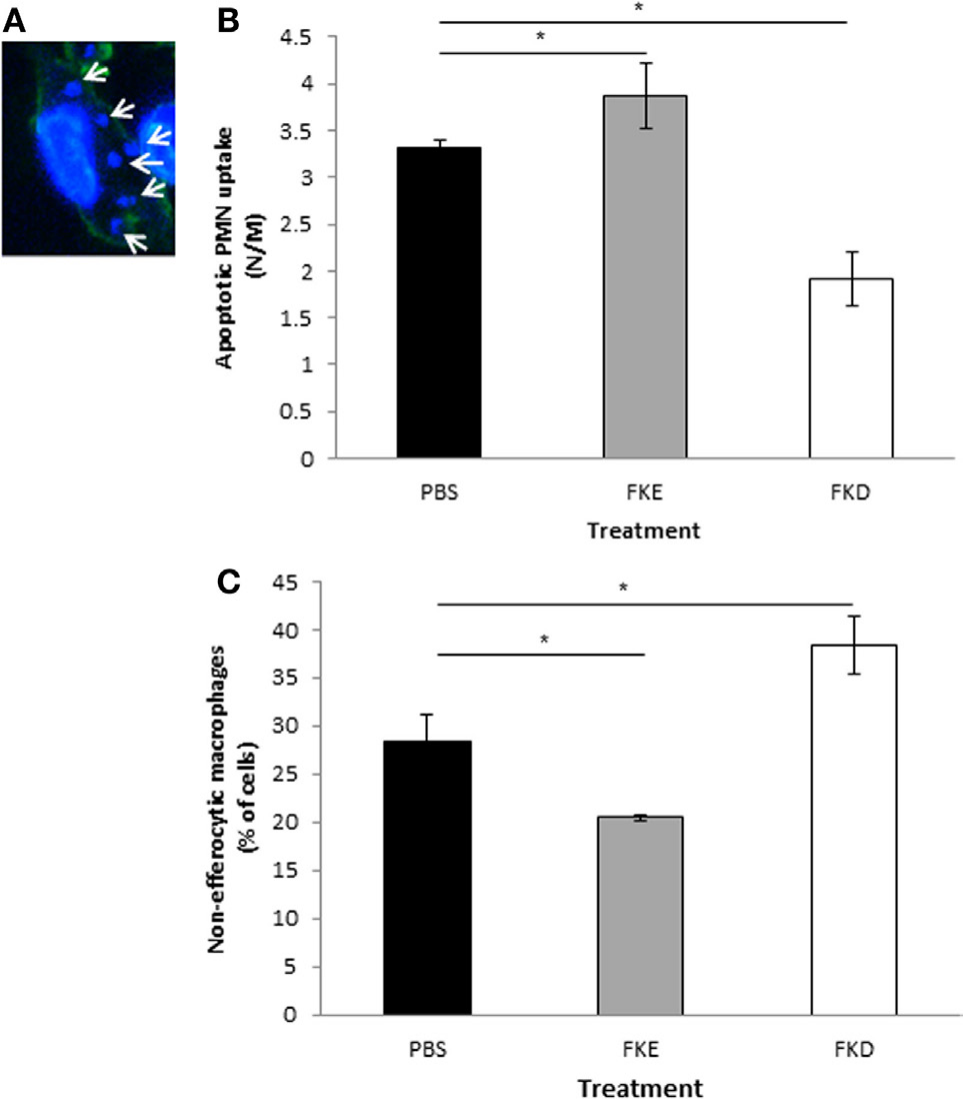
FKE peptides promote macrophage efferocytosis. Mice undergoing peritonitis for 48 h were injected intraperitoneally (i.p.) with PBS, FKE, or FKD peptides (50 µM each). After additional 18 h, peritoneal macrophages were isolated and stained with CF488A-conjugated phalloidin and Hoechst. Slides were visualized under a Nikon A1 confocal microscope and nuclei of engulfed polymorphonuclear cells (PMN) were enumerated [see **(A)** for a representative image]. Results show the average number of engulfed apoptotic PMN per macrophage **(B)** and the percentage of macrophages that did not engulf apoptotic PMN **(C)**. Results are averages ± SEM from three independent experiments (*n* = 4 per group). *indicate statistically significant differences of *P* ≤ 0.05 by ANOVA with Tukey *post hoc* analysis.

It was previously shown that macrophage conversion to the CD11b^low^ phenotype can be induced by apoptotic PMN uptake (9). Hence, we examined the impact of Lf peptides on efferocytosis *in vivo*. Our results in Figures 4A–C indicate that FKE increased efferocytosis, while FKD reduced it (1.17- and 0.57-fold of PBS, respectively). These responses were primarily due to a decrease in non-efferocytic macrophages following FKE treatment, while the percentage of these macrophages was increased by FKD (0.72- and 1.35-fold of PBS, respectively). Thus, FKE, but not FKD, peptides enhance the uptake of apoptotic PMN by resolution phase macrophages.

### FKE Peptides Promote Macrophage Reprogramming

**Figure 5:**
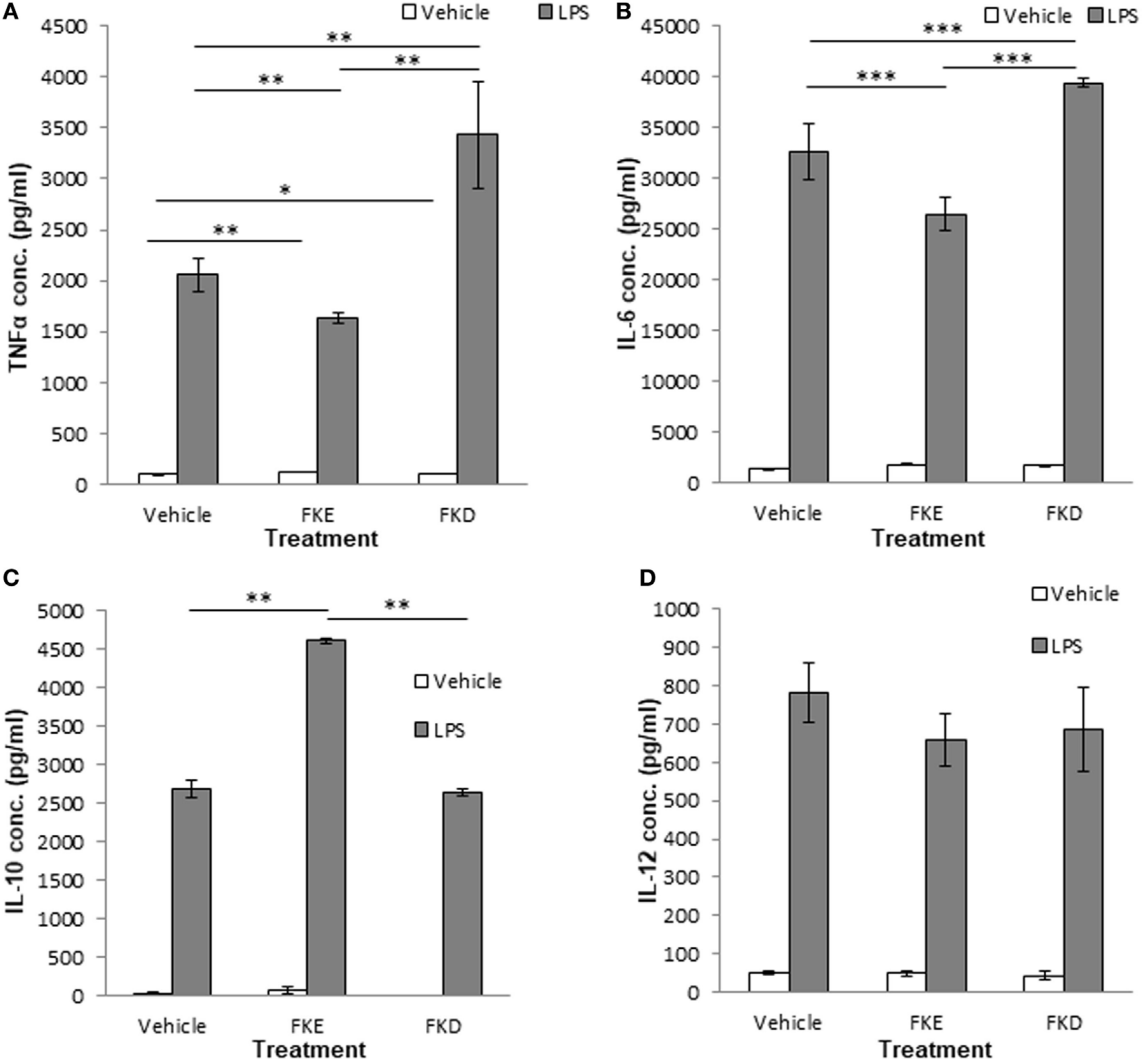
FKE peptides promote macrophage reprogramming. Mice undergoing peritonitis for 48 h were injected intraperitoneally (i.p.) with PBS, FKE, or FKD peptides (50 µM each). After additional 24 h, peritoneal macrophages were isolated and incubated *ex vivo* with lipopolysaccharide (LPS) (1 µg/ml, 24 h) or vehicle. Then, culture media were collected and its content of tumor necrosis factor α (TNFα) **(A)**, interleukin (IL)-6 **(B)**, IL-10 **(C)**, and IL-12 **(D)** was determined by standard enzyme-linked immunosorbent assay. Results are representative **(A,D)** and averages ± SEM **(B,C)** from three experiments (*n* = 4 per group). ***/**/*indicate statistically significant differences of *P* ≤ 0.005/*P* ≤ 0.01/*P* ≤ 0.05, respectively, by ANOVA with Tukey *post hoc* analysis.

The uptake of apoptotic cells promotes macrophage reprogramming and results in a reduced secretion of pro-inflammatory cytokines and an increased secretion of anti-inflammatory/reparative cytokines upon exposure to bacterial agents (5). Hence, we examined whether Lf peptides can affect macrophage reprogramming and cytokine secretion upon LPS stimulation. Our results indicate that FKE exposure *in vivo* reduced TNFα and IL-6 secretion by LPS-stimulated resolution phase macrophages [(Figures 5A,B); 0.79- and 0.81-fold of PBS, respectively]. FKD peptides, on the other hand, increased the secretion of these inflammatory cytokines from LPS-stimulated resolution phase macrophages [(Figures 5A,B); 1.66- and 1.2-fold of PBS, respectively]. Notably, IL-10 secretion was induced by FKE, but not by FKD [(Figure 5C); 1.72- and 0.98-fold of PBS, respectively], whereas both peptides reduced IL-12 secretion but not in a statistically significant manner (Figure 5D). Thus, FKE, but not FKD, seems to promote macrophage reprogramming during resolving inflammation.

### Lf-Derived Peptides Enhance MSU-Induced aggNETs Formation

**Figure 6:**
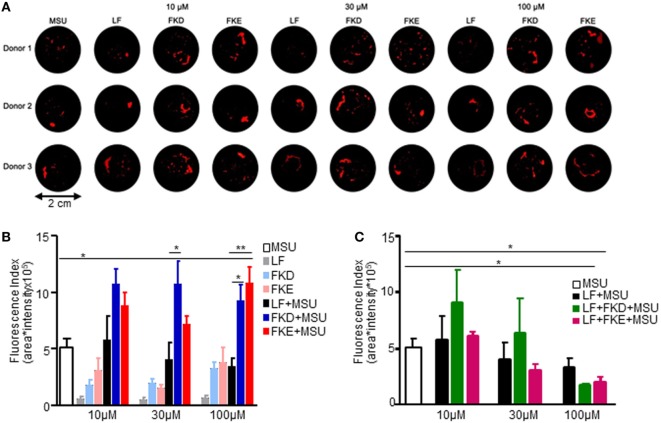
Lactoferrin (Lf)-derived peptides induce aggregated neutrophil extracellular traps (aggNETs) formation. Human polymorphonuclear cells (PMN) from three healthy donors were freshly isolated from peripheral blood and seeded in a final cell density of 5 × 10^6^ cells/ml. PMNs were incubated with 50 pg/cell monosodium urate crystals (MSU) and/or 10–100 µM Lf (LF) and/or 10–100 µM FKD or FKE peptides and 1 µg/ml propidium iodide solution. NET aggregation was stopped by fixation and aggNETs formed were filtrated through a 40-µM mesh. Macrophotographs with UVB transillumination were captured with a Nikon D700 reflex camera **(A)**. Averages ± SEM of fluorescence values from three independent donors of the images of control, MSU, and MSU + Lf **(B)** and MSU + FKD/FKE peptides at the indicated concentrations **(C)**. **/*indicate statistically significant differences of *P* ≤ 0.01/*P* ≤ 0.05, respectively, by ANOVA with Tukey *post hoc* analysis.

The resolution of neutrophilic inflammation was recently shown to be associated with the formation of cytokine-scavenging aggNETs displaying a tophus texture (21). Since Lf promotes human neutrophil aggregation at low density, we determined whether Lf, Lf peptides (LFPs), or combinations of these agents also promote aggNETs formation. To this end, we added FKE or FKD peptides alone or together with full-length Lf to MSU crystals in NETs formation and aggregation assays. Our results show that treatment with FKE enhanced MSU-induced aggNETs formation at 100 µM, whereas FKD did it to a lower and nonstatistically significant manner (Figure 6A). Full-length Lf reduced MSU-induced aggNETs formation in a concentration-dependent (but not statistically significant) manner at 30–100 µM (Figure 6B). Surprisingly, this effect was enhanced by FKE and FKD peptides (at 100 µM) in a statistically significant manner (compared to MSU alone; Figure 6B). Notably, FKD (at 30 µM) and FKE (at 100 µM) modulated MSU-induced aggNETs formation in a significantly different manner than Lf (Figure 6C), suggesting that the fragmentation of Lf is necessary for it to enhance the resolution of inflammation through aggNETs formation. We conclude that LFP enhances aggNETs formation by human neutrophils and thus contributes to the resolution of inflammation.

## Discussion

Rapid engulfment of senescent PMN by macrophages during the resolution phase of inflammation causes the accumulation of stodgy cellular remnants in phagolysosomes that is congruent with the satiation of the engulfing macrophages (9). Macrophages have developed specific molecular mechanisms to cope with this burden (28). We hypothesized that Lf, a major neutrophil constituent that contains several proteolytic peptides with variable actions, can be processed during neutrophil apoptosis into bioactive fragments that accumulate in the engulfing macrophages. These will in turn be released and regulate both inflammation and its resolution. Since macrophages are emigrating from inflammatory and resolving tissues to remote sites (9, 23, 29), they can release Lf-derived products either locally or at their final destination. Our results in Figure 1 indicate that a truncated form of Lf with 50 kDa is to be found in resolution phase macrophages and that the latter can acquire Lf from apoptotic PMN but not from engulfment of other apoptotic cells or phagocytic targets. Our results indicate that Lf and its fragments found in these macrophages are not expressed *de novo*, but rather accumulating following efferocytosis. The Lf is cleaved in either PMN or macrophages to yield smaller fragments of 23, 17, and 15 kDa, which can robustly be found in murine ISFs from spleen and inguinal LN and in the udder of cows inflicted by mastitis. Importantly, we also detected these Lf fragments in macrophages or immune cells collected at inflammatory sites. Of particular interest, the Lf fragments showed different kinetics. The 23- and 15-kDa fragments were associated with the inflammatory phase, while the 17-kDa fragment increased during the resolving phase of inflammation. Moreover, the levels of splenic 17-kDa fragment were reduced upon peritoneal macrophage depletion during the resolution phase, whereas the levels of the 23-kDa fragment were increased. Thus, apoptotic PMN and efferocytic macrophages processed Lf, and the later cells released its fragments in a spatially and temporally regulated manner during the inflammatory responses. Since the inflammatory 23- and 15-kDa fragments of Lf were not found in apoptotic PMN, it is not clear whether they are generated by diversion of the proteolytic cascade in PMN that were engulfed, the efferocytic macrophages themselves or cleavage of secreted Lf at local or remote sites.

Notably, during bacterial infection, a Lf fragment with 22 kDa was previously described in bovine and human samples (19, 20). This fragment contained four peptides generated by the serine proteases elastase and proteinase 3. One of these peptides with the sequences PGQRDLLFKDSAL/SGQKDLLFKDSAI in bovine and human Lf, respectively, induced cytokine and chemokine secretion in epithelial cells. Another peptide present in human Lf, FKDCHLA, induced inflammatory cytokine secretion, while its bovine homolog FKECHLA was inactive. It seems that the FKD tripeptide present in the first three peptides might account for their activity while the replacement of aspartic acid (D) to glutamic acid (E) results in the abortion of the stimulatory activity. Hence, we examined whether FKE or FKD peptides can shift resolution indices *in vivo*. Our previous studies (9) showed that a distinct subset of pro-resolving macrophages designated CD11b^low^ macrophages were converted from CD11b^high^ ones upon satiated efferocytosis of apoptotic PMN. These macrophages produced reduced levels of inflammatory cytokines and increased levels of tumor growth factor β (TGFβ) in comparison to their CD11b^high^ counterparts. Earlier studies have shown that the uptake of apoptotic cells by monocyte/macrophage leads to their reprogramming that is accompanied by a reduced inflammatory cytokine production and increased IL-10 and TGFβ secretion (30, 31). Our current results indicate that FKE, and to a lesser degree FKD, reduced macrophage numbers in the peritoneum while increasing the percentage of CD11b^low^ macrophages (Figure 3). In addition, FKE peptides promoted efferocytosis of apoptotic PMN (Figure 4) and reduced TNFα and IL-6 secretion while increasing IL-10 secretion by resolution phase macrophages in response to LPS, while FKD peptides exerted opposite actions (Figure 5). Altogether, our findings suggest that peptides present within the resolution-associated fragment of Lf can modulate resolution phase macrophage reprogramming toward a pro-resolving phenotype, with FKE having superior actions to FKD.

**Figure 7:**
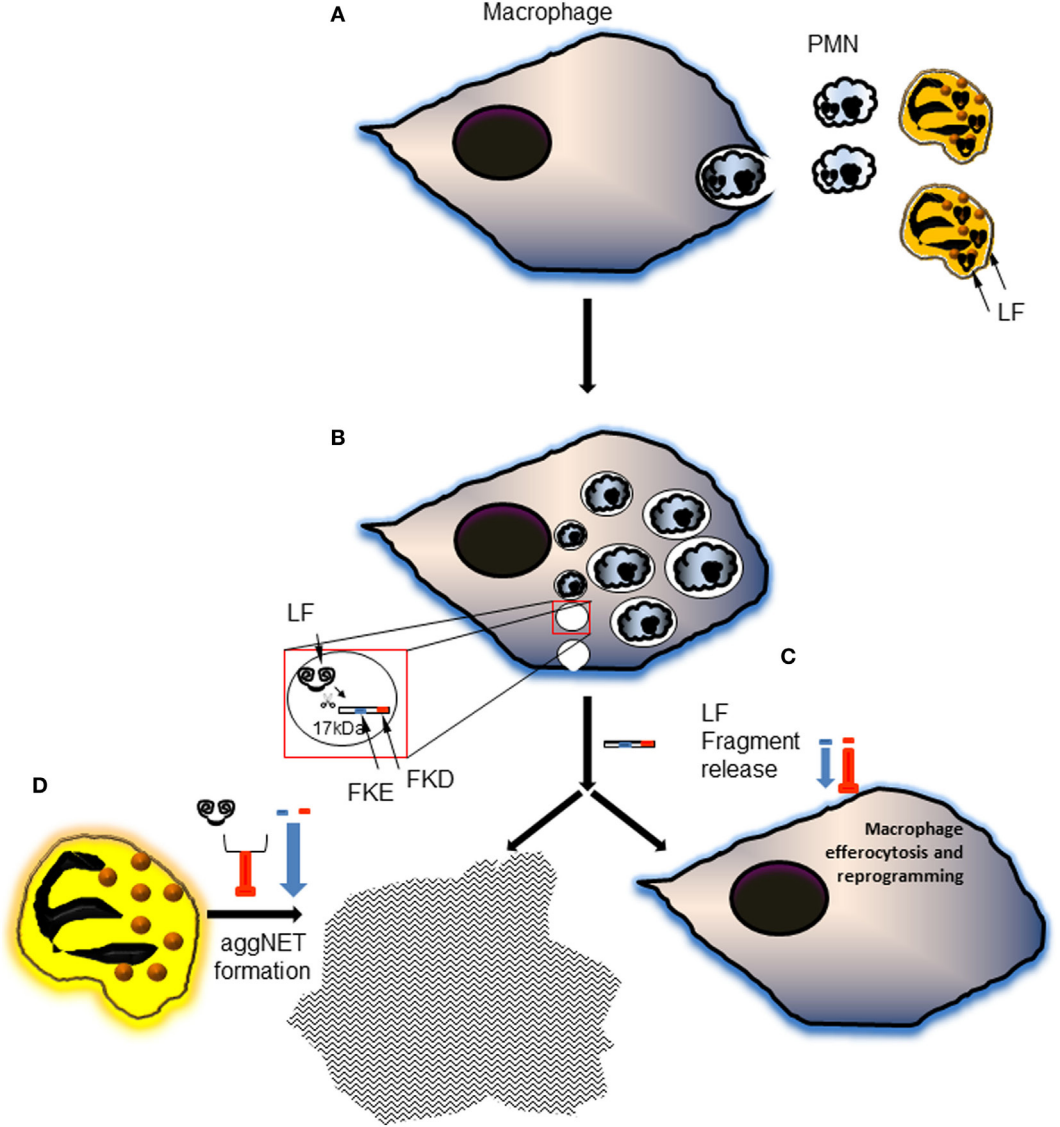
A schematic illustration of the proposed model of generation and action of lactoferrin (Lf) fragments and peptides during the resolution of inflammation. **(A)** During inflammation and its resolution, neutrophils undergo apoptosis and get engulfed by macrophages. This process is accompanied by neutrophil elastase-mediated cleavage of Lf and generation of the 17-kDa fragment. **(B)** The engulfed polymorphonuclear cell (PMN) corpses traffic to the phagolysosome where most of their cellular components are hydrolyzed. Lf fragments are accumulating in resolution phase macrophages during the inflammatory and resolving phases of the response. 15- and 17-kDa fragments were produced during the inflammatory phase and the resolution of inflammation, respectively. The 17-kDa Lf fragment is released and possibly undergoes further processing to shorter peptides containing the tripeptides FKE and FKD. **(C)** Both peptides enhance macrophage conversion to the CD11b^low^ phenotype, whereas FKE peptides enhance macrophage efferocytosis *in vivo*. FKE peptides also promote macrophage reprogramming *in vivo* culminating in a reduced secretion of tumor necrosis factor α (TNFα) and interleukin (IL)-6, and an increased secretion of IL-10 upon exposure to LPS. **(D)** Lf peptides also promote the generation of pro-resolving aggregated neutrophil extracellular traps (aggNETs) in monosodium urate (MSU)-activated human neutrophils, while Lf fails to do so. Intriguingly, when combined with full-length Lf, Lf peptides enhance its inhibition of aggNETs formation following MSU exposure. Altogether, these findings suggest that Lf undergoes specific proteolytic processing to yield functional fragments that are released and “recycled”. These fragments contain bioactive peptides with anti-inflammatory and pro-resolving properties that orchestrate the resolution of neutrophil-driven inflammation.

Lactoferrin has previously been shown to enhance MSU-induced aggregation of neutrophils at a low cellular density in PBS (21), but to limit phorbol myristate acetate-induced NET formation due to its positively charged amino acids (32). These apparently conflicting results can be explained by the activity of Lf in different *in vitro* conditions. In the presence of culture media like RPMI, full-length Lf has no effect on the formation of aggNETs induced by MSU crystals. Importantly, the FKE peptide, and to a lesser degree FKD, enhanced the pro-resolving action of MSU in promoting aggNETs formation even in low neutrophil densities (Figure 6). Both peptides had a significantly different impact than the parent protein, but surprisingly exerted an inhibitory effect on aggNETs formation when applied together with full-length Lf. Thus, LFP promoted human neutrophil-mediated resolution of inflammation by enhancing the generation of aggNETs, while full-length Lf failed to do so. These findings suggest that Lf cleavage and disposal of its positive charge are essential for its promotion of aggNETs formation (see Figure 7 for illustration).

In sum, the current report unveils the generation and release of Lf-derived fragments temporally changing during the initiation and resolution of inflammation. The fragment that is prevalent during the resolution phase contains bioactive peptides that promote pro-resolving actions of human neutrophils and macrophages most likely through binding to surface receptors and intracellular signaling. These findings improve our understanding of the molecular aspects of resolving inflammation and may lead to novel Lf-based therapies for inflammatory conditions.

## Data Availability Statement

The mass spectrometry proteomics data have been deposited to the ProteomeXchange Consortium *via* the PRIDE [1] partner repository with the dataset identifier PXD009190.

## Ethics Statement

Experiments were approved by the Committee of Ethics, The Technion, authorization no. IL-065-04-2010.

## Author Contributions

AL isolated macrophages and ISF from murine peritonitis, and protein extracts from milk samples, *in vivo* LFP experiments, performed flow cytometry analysis, and drafted the manuscript. SS performed clodronate depletion, isolated fluids and cells, analyzed samples by flow cytometry and Western blotting, stained and scored for efferocytosis, and measured cytokine secretion. SS-Z assisted in various aspects of the experimental design and experimentation. OT performed Western blotting of milk samples. RR performed the senescent PMN engulfment assays. LM and MP performed the aggNETs experiments. LM, MH, and CS assisted in designing the aggNETs experiments and analyzing the results. AA designed the study, assisted in analyzing the data, and wrote the manuscript.

## Conflict of Interest Statement

The authors declare a potential conflict of interest and state it below. Patent applications in which AA, AL, and SS-Z are inventors were approved for the data presented in the manuscript: 1. Lactoferrin Fragments And Use Thereof (US 20140162377). 2. Synthetic Anti-Inflammatory Peptides and Use Thereof (WO 2014174517A1).

## Footnotes

^1^www.bioinformatics.org/strap/.

^2^www.ncbi.nlm.nih.gov/pubmed/.

## Supplementary Material

Figure S1 Senescent peritoneal neutrophils are primarily apoptotic with CXCR4 expression. Mice were injected with zymosan A (1 mg/mouse, i.p.), and neutrophils were isolated from peritoneal exudates at 4 h post-peritonitis initiation (PPI), and incubated with roscovitine (10 µM) or vehicle for 4–24 h. Next, the neutrophils were immunostained for FITC-annexin V and PE-CXCR4 and analyzed by flow cytometry. Results are representative dot plots **(A)** and averages ± SEM **(B)** from five to six independent experiments. ***/*indicate statistically significant differences of *P* ≤ 0.005/*P* ≤ 0.05, respectively, by ANOVA with Tukey *post hoc* analysis.

Figure S2 Lactoferrin (Lf) fragment isolation and identification. Mastitis milk samples from dairy farms **(A,B)** were processed to separate the cellular and fat fractions from the aqueous fraction [**(C–F)**, see Section “Materials and Methods” for details] and to prepare them for isolation of Lf peptides. To reduce the amount of low-molecular weight constituents, the samples were dialyzed (cutoff of 12–14 kDa). Then, Lf fragments were isolated on heparin columns **(G,H)**. After stepwise elution with increasing NaCl concentrations, the samples were run on sodium dodecyl sulfate-polyacrylamide gel electrophoresis (SDS-PAGE) and the gel was stained with Coomassie Blue **(I)**. Afterward, the 15- and 17-kDa bands were excised, digested, and analyzed by MS-MS mass spectrometry **(J,K)**. The peptides contained in the fragments were identified and aligned in comparison to full-length Lf from various species **(L)**. Adding quantitative properties of the MS-MS analysis and the known immunogenic epitope in both fragments allowed the determination of the fragment boundaries. The alignment of the various Lf species and the reports by Komine et al. allowed us to predict potentially bioactive peptides present in the 17-kDa fragment of bovine Lf. These peptides were synthesized and purchased from GL Biochem (Shanghai) Ltd. **(M)**.

Figure S3 The 17- and 15-kDa fragments of lactoferrin (Lf) are absent in non-resolving and healthy dairy cow milk. Milk samples from none-resolving mastitis-inflicted dairy cows (days 1–4) or healthy (H) cows were separated into soluble and cellular fractions by centrifugation. The cellular fraction was lysed, and the cytoplasmic proteins were recovered. Equal amounts of protein from both fractions were analyzed by Western blotting for Lf. Results show representative blots from two sample sets taken daily from two different cows.

Figure S4 Alignment of the identified lactoferrin (Lf)-derived fragments to full-length Lf originated from different species. Protein bands corresponding to the 15- and 17-kDa fragments of Lf were sequenced using trypsin cleavage and mass spectrometry analysis. The sequences of the resulting peptides were aligned and compared to full-length Lf from nine species: *Capra hircus, Equus caballus, Ovis aries, Sus scrofa, Camelus dromedarius, Bos indicus, Bos taurus, Homo sapiens*, and *Mus musculus*, using STRAP software. Highlighted in yellow and in bright purple are peptides corresponding to the 15- and 17-kDa fragments, respectively. Positions 1–19 of all sequences (excluding *E. caballus*) serve as a signal peptide, and therefore amino acid numbering starts from the 19th amino acid. Peptides produced by serine proteases and reported by Komine et al. (FKECHLA, VPSHAVVAR, FQLFGSP, and PGQRDLLFKDSAL from bovine Lf) are underlined. The sequences in the red frame indicate the minimal consensus sequences in the 17-kDa fragment that were predicted to be bioactive.

## Abbreviations

aggNETs, aggregated neutrophil extracellular traps; ATP, adenosine triphosphate; BMEC, bovine mammary gland epithelial cells; CID, collision-induced dissociation; ELISA, enzyme-linked immunosorbent assay; FCS, fetal calf serum; IgG, immunoglobulin G; IL, interleukin; ISF, interstitial fluid; LBs, latex beads; Lf, lactoferrin; LFPs, lactoferrin peptides; LPS, lipopolysaccharide; LN, lymph nodes; MCP-1, monocyte chemotactic protein-1; NF-κB, nuclear factor kappa B; PMA, phorbol myristate acetate; PMN, polymorphonuclear cells; PPI, post-peritonitis initiation; PVDF, polyvinylidene difluoride; RIPA, radioimmunoprecipitation assay; ROS, reactive oxygen species; SDS-PAGE, sodium dodecyl sulfate-polyacrylamide gel electrophoresis; TGFβ, tumor growth factor β; TNFα, tumor necrosis factor α.
