# Supplementary figures and images for: A 17-kDa Fragment of Lactoferrin Associates With the Termination of Inflammation and Peptides Within Promote Resolution
Source: Front Immunol. 2018 Mar 28;9:644. doi: 10.3389/fimmu.2018.00644 (PMC5882790; doi:10.3389/fimmu.2018.00644)

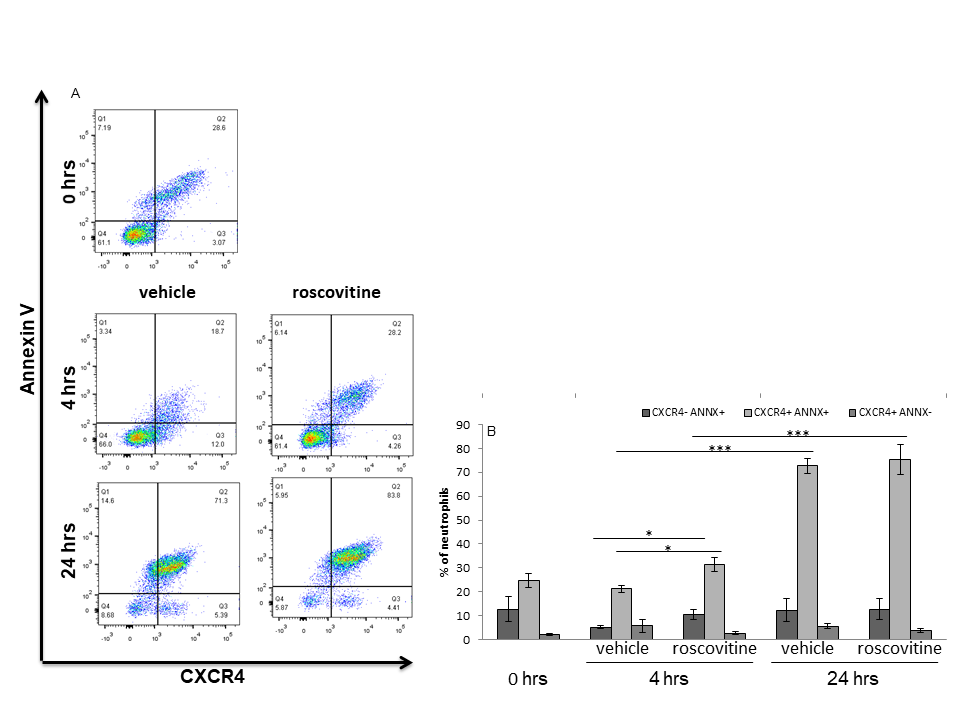

Supplement: Figure S1 — Senescent peritoneal neutrophils are primarily apoptotic with CXCR4 expression. Mice were injected with zymosan A (1 mg/mouse, i.p.), and neutrophils were isolated from peritoneal exudates at 4 h post-peritonitis initiation (PPI), and incubated with roscovitine (10 µM) or vehicle for 4–24 h. Next, the neutrophils were immunostained for FITC-annexin V and PE-CXCR4 and analyzed by flow cytometry. Results are representative dot plots (A) and averages ± SEM (B) from five to six independent experiments. ***/*indicate statistically significant differences of P ≤ 0.005/P ≤ 0.05, respectively, by ANOVA with Tukey post hoc analysis. [file image_1.tif]

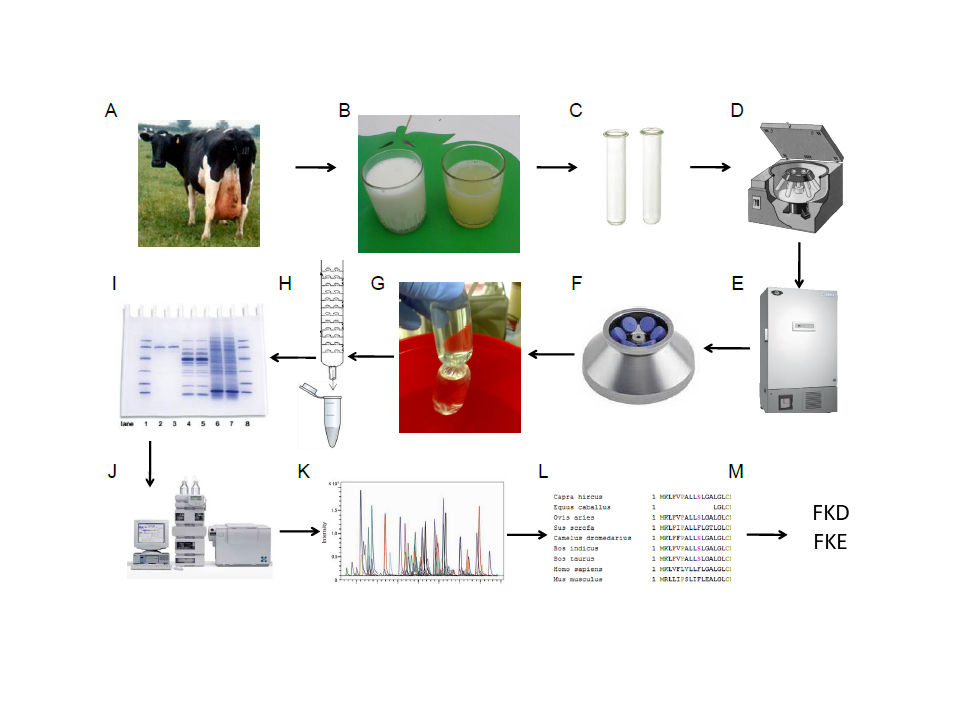

Supplement: Figure S2 — Lactoferrin (Lf) fragment isolation and identification. Mastitis milk samples from dairy farms (A,B) were processed to separate the cellular and fat fractions from the aqueous fraction [(C–F), see Section “Materials and Methods” for details] and to prepare them for isolation of Lf peptides. To reduce the amount of low-molecular weight constituents, the samples were dialyzed (cutoff of 12–14 kDa). Then, Lf fragments were isolated on heparin columns (G,H). After stepwise elution with increasing NaCl concentrations, the samples were run on sodium dodecyl sulfate-polyacrylamide gel electrophoresis (SDS-PAGE) and the gel was stained with Coomassie Blue (I). Afterward, the 15- and 17-kDa bands were excised, digested, and analyzed by MS-MS mass spectrometry (J,K). The peptides contained in the fragments were identified and aligned in comparison to full-length Lf from various species (L). Adding quantitative properties of the MS-MS analysis and the known immunogenic epitope in both fragments allowed the determination of the fragment boundaries. The alignment of the various Lf species and the reports by Komine et al. allowed us to predict potentially bioactive peptides present in the 17-kDa fragment of bovine Lf. These peptides were synthesized and purchased from GL Biochem (Shanghai) Ltd. (M). [file image_2.tif]

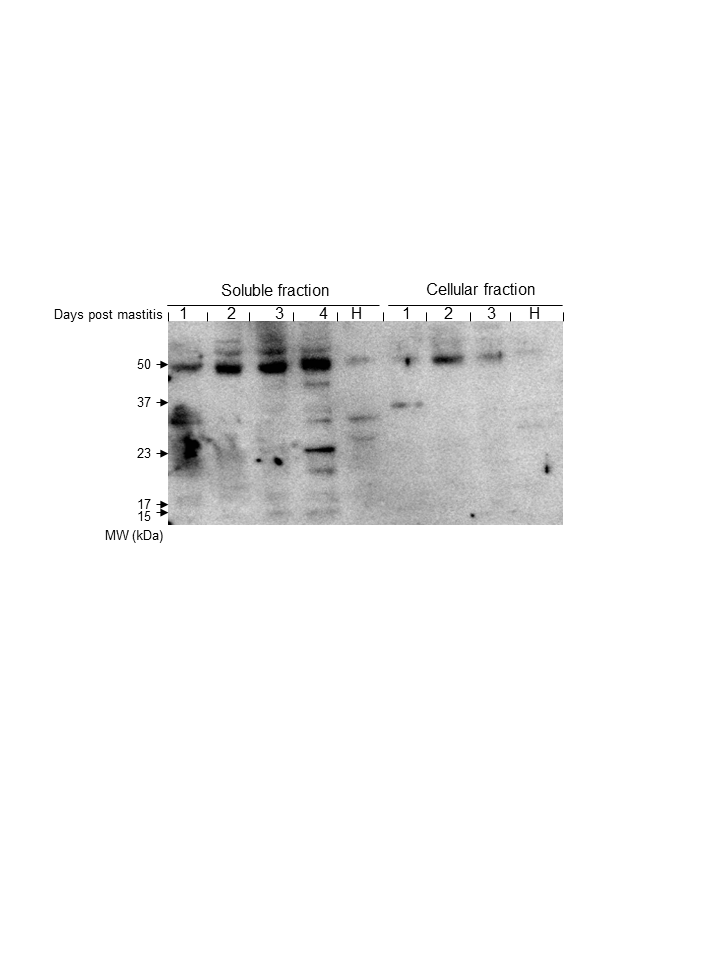

Supplement: Figure S3 — The 17- and 15-kDa fragments of lactoferrin (Lf) are absent in non-resolving and healthy dairy cow milk. Milk samples from none-resolving mastitis-inflicted dairy cows (days 1–4) or healthy (H) cows were separated into soluble and cellular fractions by centrifugation. The cellular fraction was lysed, and the cytoplasmic proteins were recovered. Equal amounts of protein from both fractions were analyzed by Western blotting for Lf. Results show representative blots from two sample sets taken daily from two different cows. [file image_3.tif]

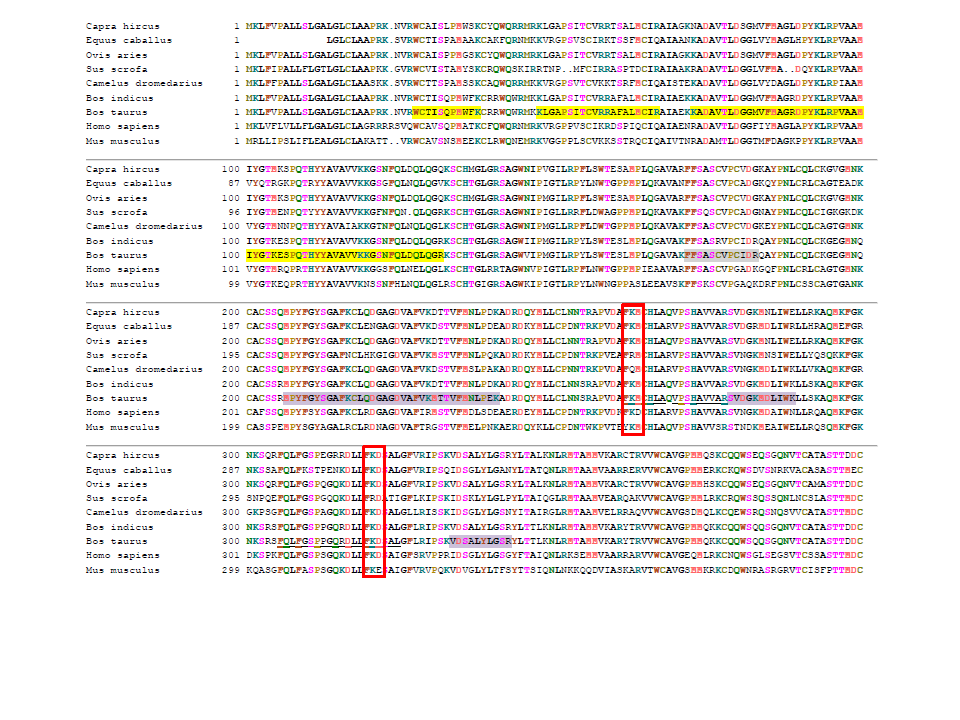

Supplement: Figure S4 — Alignment of the identified lactoferrin (Lf)-derived fragments to full-length Lf originated from different species. Protein bands corresponding to the 15- and 17-kDa fragments of Lf were sequenced using trypsin cleavage and mass spectrometry analysis. The sequences of the resulting peptides were aligned and compared to full-length Lf from nine species: Capra hircus, Equus caballus, Ovis aries, Sus scrofa, Camelus dromedarius, Bos indicus, Bos taurus, Homo sapiens, and Mus musculus, using STRAP software. Highlighted in yellow and in bright purple are peptides corresponding to the 15- and 17-kDa fragments, respectively. Positions 1–19 of all sequences (excluding E. caballus) serve as a signal peptide, and therefore amino acid numbering starts from the 19th amino acid. Peptides produced by serine proteases and reported by Komine et al. (FKECHLA, VPSHAVVAR, FQLFGSP, and PGQRDLLFKDSAL from bovine Lf) are underlined. The sequences in the red frame indicate the minimal consensus sequences in the 17-kDa fragment that were predicted to be bioactive. [file image_4.tif]
